# Functional regulation of YAP mechanosensitive transcriptional coactivator by Focused Low-Intensity Pulsed Ultrasound (FLIPUS) enhances proliferation of murine mesenchymal precursors

**DOI:** 10.1371/journal.pone.0206041

**Published:** 2018-10-26

**Authors:** Regina Puts, Paul Rikeit, Karen Ruschke, Petra Knaus, Sophie Schreivogel, Kay Raum

**Affiliations:** 1 Berlin-Brandenburg Center for Regenerative Therapies (BCRT), Charité–Berlin University of Medicine, Berlin, Germany; 2 Berlin-Brandenburg School for Regenerative Therapies (BSRT), Charité–Berlin University of Medicine, Berlin, Germany; 3 Institute of Chemistry and Biochemistry, Free University of Berlin, Berlin, Germany; 4 Julius Wolff Institute, Charité–Berlin University of Medicine, Berlin, Germany; University of Colorado Boulder, UNITED STATES

## Abstract

Yes-associated protein (YAP) acts as a mechanotransducer in determining the cell fate of murine C2C12 mesenchymal precursors as investigated after stimulation with ultrasound. We applied Focused Low-Intensity Pulsed Ultrasound (FLIPUS) at a sound frequency of 3.6 MHz, 100 Hz pulse repetition frequency (PRF), 27.8% duty cycle (DC), and 44.5 mW/cm^2^ acoustic intensity I_SATA_ for 5 minutes and evaluated early cellular responses. FLIPUS decreased the level of phosphorylated YAP on Serine 127, leading to higher levels of active YAP in the nucleus. This in turn enhanced the expression of YAP-target genes associated with actin nucleation and stabilization, cytokinesis, and cell cycle progression. FLIPUS enhanced proliferation of C2C12 cells, whereas silencing of YAP expression abolished the beneficial effects of ultrasound. The expression of the transcription factor MyoD, defining cellular myogenic differentiation, was inhibited by mechanical stimulation. This study shows that ultrasound exposure regulates YAP functioning, which in turn improves the cell proliferative potential, critical for tissue regeneration process.

## Introduction

Together with biochemical cues, the mechanical environment experienced by cells heavily influences proper development and function of organ-forming tissues. For example, the differentiation potential of Mesenchymal Stem Cells (MSCs) can be directed into various tissues solely by alteration of the substrate stiffness [1], tumor growth and progression decelerates by softening of the microenvironment [2], and cellular proliferation can be enhanced with the stiffening of the carrier matrix [3]. Normal tissue homeostasis highly depends on mechanical forces acting on the cells [4]. However, the mechanisms converting the mechanical inputs experienced by the cell into a biochemical response and the involved mechanotransduction signaling pathways are currently only partially understood. The discovery of the mechanosensitive properties of Yes-associated Protein (YAP) and its structural homologue transcriptional co-activator with PDZ-binding motif (TAZ) has shed some light on the mechanistic events directing cellular commitment in response to mechanical stimuli [3, 5].

YAP was first isolated as a binding partner of Yes-protein-tyrosine kinase [6] and later found to be a nuclear effector of the Hippo signaling pathway, controlling organ homeostasis and tissue regeneration [7]. In its active state, YAP is localized in the nucleus and regulates a variety of cellular processes such as proliferation, apoptosis, and migration, generally promoting cell division and inhibiting differentiation of cells. Since YAP itself lacks a DNA-binding domain, it binds to a number of transcription factors to exert its functions, with TEAD family proteins being the most common partners [8–10]. Activation of the Hippo cascade by various biochemical or mechanical triggers leads to YAP phosphorylation and therefore inactivation. Most prominently, phosphorylation at Serine 127 leads to binding to cytosolic 14-3-3 proteins, resulting in cytoplasmic retention of YAP, terminating its nuclear activity [7].

YAP activity was originally found to be regulated by contact inhibition of proliferation [7]. In 2011, Dupont *et al*. [3] demonstrated that mechanotransduction *via* YAP is dependent on cell geometry and cytoskeletal tension: cells being able to spread over a large area exhibit active nuclear YAP, whereas a confined cellular morphology leads to retention of YAP in the cytoplasm. This was later supported by a study of Aragona *et al*. [5], who reported that in densely seeded epithelial cells, YAP translocates to the nucleus when the monolayer is stretched, resulting in faster proliferation compared to unstretched cells.

Cellular morphology and tension are shaped by the cytoskeleton, i.e., through the dynamics of contractile actomyosin filaments [11]. A direct link has been established between YAP activity and the presence of actin stress fibers. Thick fibers of filamentous actin (F-actin) promote nuclear accumulation of YAP, whereas inhibitors of actin polymerization or myosin activity, latrunculin A and blebbistatin, respectively, ablate YAP activity [12]. Inhibition of actin-severing proteins, such as Cofilin, Gelsolin, and CapZ, leads to F-actin bundle formation, thus, increasing YAP activity [5]. Treatment of cells with the actin stabilizing drug jasplakinolide drives YAP into the nucleus [13].

Throughout its lifetime a cell can experience a range of mechanical stimuli that influence its fate, including compression, tension, hydrostatic pressure, and shear force [14]. Low-Intensity Pulsed Ultrasound (LIPUS) is a mechanical technique eliciting complex physical phenomena, which is used in clinical praxis for bone healing [15] and holds a potential for regeneration of soft tissues [16]. However, the mechanisms leading to the observed regenerative effects still remain contentious [17]. The focused LIPUS (FLIPUS) used in our experiments represents a well-characterized *in-vitro* set-up, enabling quantification of the mechanical dose by minimizing the introduced physical artifacts, i.e., standing waves, temperature elevations, and near-field interferences [18], usually associated with *in-vitro* devices employing planar transducers [17]. We have previously demonstrated that FLIPUS induces transcriptional mechanoresponses in murine C2C12 mesenchymal precursor cells *via* the activation of AP-1, Sp1, and TEAD transcription factors [19]. Due to the tight connection in co-transcriptional activity between TEAD and YAP, in the current study we investigated whether YAP is a key player transducing the FLIPUS mechanical signal into the nucleus and regulating the proliferation of C2C12 cells in response to the spatial average temporal average intensity I_SATA_ = 44.5 mW/cm^2^.

## Materials and methods

### FLIPUS *in-vitro* cell stimulation set-up

The FLIPUS cell-stimulation set-up was calibrated and described elsewhere [18]. Briefly, the set-up consists of an array of four focused transducers positioned at the bottom of a temperature-controlled (37°C) water tank, which is filled with deionized degassed water. The 24-well-plate, carrying C2C12 cell-monolayers, was submerged in the water above the transducer array, so that the diverging defocused far-field of each of the four probes overlapped with the area of a well. The set-up enabled homogenous intensity distribution within the well, without introduction of unwanted physical effects, i.e., temperature elevations and standing waves. The cells in the chamber were supplied with 5% CO_2_ and 95% air mixture. In our experimental set-up, C2C12 cells were seeded at 9*10^4^ cells/well density (if not mentioned otherwise) in order to maintain a constrained circular cellular morphology. Forty-eight hours later the cells were starved for 3 h in the expansion media (see below) without fetal calf serum (FCS) and stimulated for 5 min with FLIPUS. Each individual time point had its own negative control, which were cultured in the same well-plate, but did not receive FLIPUS exposure. The FLIPUS signal consisted of 3.6 MHz excitation frequency, 100 Hz pulse repetition frequency, 27.8% duty cycle and spatial average temporal average intensity I_SATA_ = 44.5 mW/cm^2^, corresponding to the 89 kPa, average mean peak pressure amplitude. This intensity was previously used to enhance activity of TEAD transcription factor [19], a binding partner of YAP.

### Cell culturing

Murine C2C12 mesenchymal precursors were obtained from ATCC. The cells were expanded in Dulbecco Modified Eagle’s Medium (DMEM), supplemented with 10% FCS, 1% Penicillin/Streptomycin (Biochrom AG, Berlin, Germany), 2 mM GlutaMaxTM (GIBCO, Life Technologies, Darmstadt, Germany) under standard cultivation conditions (37°C, 5% CO2, 95% humidity).

### siRNA mediated YAP knock-down

C2C12 cells were seeded at a density of 5*10^4^ cells/well. Twenty-four hours later, cells were transfected with either siRNA targeting YAP (SMARTpool ON-TARGETplus YAP1, Dharmacon, Lafayette, CO, USA), or scrambled control siRNA (ON-TARGET plus Non-targeting Control siRNA #1, Dharmacon, Lafayette, CO, USA), from now on referred to as siYAP and siScr, respectively, at a final concentration of 50 nM per well. The siRNAs were mixed with 2.5 μl Lipofectamine RNAiMAX (Invitrogen, ThermoFisher Scientific, Waltham, MA, USA) transfection reagent per well. The next day the cells were starved for 3 h, stimulated for 5 min with FLIPUS, and analyzed by BrdU-ELISA as described below.

Additionally, several genes were selected as potential YAP targets (see the primers’ list below) and their expression was quantified by qRT-PCR in both siYAP and siScr samples.

### BrdU-ELISA

After FLIPUS treatment, the cells were labeled for 12 h with BrdU solution. The BrdU-ELISA was performed after the labeling according to the manufacturer’s protocol (Roche Diagnostics GmbH, Mannheim, Germany). The amount of the incorporated BrdU was then quantified colorimetrically. Absorbance values were measured at 450 nm with a reference to 690 nm wavelength on TECAN infinite, M200 PRO (Maennedorf, Switzerland).

### Western blotting

Cells were lysed 0 h, 1 h, 2 h, 3 h, 4 h, and 5 h after the sonication with 2X Laemmli Sample Buffer. The cell lysates were separated on 12.5% polyacrylamide gels and transferred for 2 h to a nitrocellulose membrane with 0.45 μm pore-size (neoLab Migge GmbH, Heidelberg, Germany). The membranes were blocked in TBST buffer (TBS buffer with 0.1% Tween-20 (Sigma-Aldrich, Munich, Germany)) at pH 8, containing 3% Bovine Serum Albumin (BSA) (Carl Roth GmbH + Co. KG, Karlsruhe, Germany) for 1 h at room temperature. Then the membranes were incubated overnight at 4°C in either rabbit anti-phospho-YAP(Serine127) (Cell Signaling Technology, Inc., Danvers, MA, USA), or rabbit anti-GAPDH (Cell Signaling Technology, Inc., Danvers, MA, USA), or mouse anti-YAP (Santa Cruz Biotechnology, Dallas, TX, USA) antibody. The next day the membranes were washed 3 times in TBST buffer and incubated for 50 min at room temperature in TBST buffer containing 3% BSA and either IRDye® 800CW Goat (polyclonal) Anti-Rabbit IgG (H+L) or 680RD Goat (polyclonal) Anti-Mouse IgG (H+L), Highly Cross Adsorbed secondary antibody (LI-COR, Lincoln, NE, USA). The membranes were washed twice with TBST and once with TBS buffer. The near-infrared fluorescence signal was imaged and quantified by LI-COR Odyssey system.

### Immunofluorescence staining

C2C12 cells were seeded at a density of 5*10^4^ cells/well. The next day the cells were starved for 3 h in the expansion media containing no FCS and stimulated for 5 min with FLIPUS. Two hours after the stimulation the cells were fixed in 4% paraformaldehyde solution. The cells were rinsed with phosphate buffered saline, PBS (Biochrom AG, Berlin, Germany) and permeabilized for 30 min in 0.1% Triton^TM^ X-100 (Sigma Aldrich, Steinheim, Germany). This was followed by blocking the cells in PBS containing 3% BSA (Carl Roth GmbH + Co. KG, Karlsruhe, Germany) for 1 h. After the blocking step, primary mouse anti-YAP antibody (Santa Cruz Biotechnology, Dallas, TX, USA, 1:300) was diluted in PBS containing 1% BSA, added to the fixed cells, and incubated for 1 h. For YAP staining, the cells then were rinsed with PBS 3 times and incubated for 1 h in secondary Alexa Fluor 594 goat anti-mouse (H+L) antibody (Thermo Fischer Scientific, Rockford, IL, USA) at 1:150 ratio, prepared in PBS with 1% BSA. Until this point the procedure was carried out at room temperature. Next, the cells were rinsed again and stained with 5 μM Hoechst 33342 (Thermo Fischer Scientific, Eugene, OR, USA) solution for 20 min at 37°C. Traces of the staining were removed by PBS and the cells were mounted in Flouromount-G^®^ (Southern Biotech, Birmingham, AL, USA) medium. The stainings were visualized with Zeiss Axio Observer Z1. For YAP subcellular localization analysis, microscopy pictures from three independent experiments were analyzed by blind counting of cells with predominantly cytosolic, nuclear, or equally distributed YAP staining. 2838 non-sonicated cells, 2604 FLIPUS-treated cells, and 1437 FSC stimulated cells were counted. The ImageJ plugin of Dr. Kurt De Vos from the University of Sheffield was used to assist with the cell counting.

Histone H3 is phosphorylated on Ser28 during chromosomal condensation in mitotic phase of cell cycle. To evaluate the number of cells undergoing mitosis, the procedure described above was also applied for immunofluorescence analysis of phospho-histone H3 on Ser28. The cells fixed after the starvation were treated according to the procedure described above, with few changes made: anti-phospho-Histone H3 (Ser28) antibody (Cell Signaling Technology, Frankfurt, Germany) diluted 1:400 and then in Alexa Fluor 488 goat anti-rabbit (H+L) antibody (Thermo Fischer Scientific, Rockford, IL, USA) diluted 1:40 were used. Five pictures were taken within each well for three biological trials. The cells positively stained for p-Histone H3 (Ser28) and total cell number were then counted providing the percentage of cells undergoing mitosis.

### Cell fractionation assay

Two hours after 5 min of FLIPUS exposure, the cells were scraped off the culture dish and collected in 600 μl PBS. Subcellular fractions were obtained using NE-PER^TM^ Nuclear and Cytoplasmic Extraction Reagents (Thermo Fischer Scientific, Darmstadt, Germany) according to the manufacturer’s protocol. The western blotting procedure was performed as described above with few modifications. Rabbit anti-Histone H3 antibody (Cell Signaling Technology, Inc., Danvers, MA, USA) was used for loading controls in the nuclear fractions. Horseradish peroxidase-conjugated goat-anti-mouse IgG + IgM [H+L] and goat-anti-rabbit IgG [H+L] secondary antibodies (Dianova GmbH, Hamburg, Germany) were used for detection. The antibodies were diluted at 1:1000 ratio in TBST containing 3% BSA and the membranes were incubated for 1 h at room temperature. WesternBright Quantum HRP substrate (Advansta Inc., Menlo Park, USA) was added and signals were detected on Fusion Fx-7 Imager (Vilber Lourmat, Eberhardzell, Germany). Densitometric quantification of bands was performed using ImageJ software.

### RNA extraction and quantitative Real Time PCR (qRT-PCR)

Cells were lysed 0 h, 1 h, 3 h, 5 h, and 7 h after FLIPUS stimulation. mRNA isolation was performed using the NucleoSpin RNA II Kit (Machery Nagel, Düren, Germany). The mRNA was transcribed to complementary DNA (cDNA) with help of qScriptTM cDNA SuperMix (Quanta Biosciences, Gaithersburg, MD, USA) using Mastercycler EP Gradient S (Eppendorf, Hamburg, Germany). The quantities of cysteine-rich angiogenic inducer 61 (Cyr61), CyclinD1, amphiregulin (AREG), anillin (ANLN), diaphanous 1 and 3 formins (Diaph1 and Diaph3), Ras-homolog gene family, member A (RhoA), Rho-associated coiled-coil containing protein kinase 1 (Rock1), cell division controlling homolog 42 (CDC42), Ras-related C3 botulinum toxin substrate 1 (Rac1), connective tissue growth factor (CTGF), myogenic factor 3 (MyoD) and YAP were measured using PerfeCTa®SYBR®Green SuperMix (Quanta Biosciences, Gaithersburg, USA) on LightCycler 480 II (Roche Diagnostics GmbH, Mannheim, Germany). The primers were designed by Primer3 Input software (Version 0.4.0) and ordered from TIB Molbiol (Berlin, Germany). The expression of all target genes was normalized to the expression of a house-keeping gene hypoxanthine-guanine phosphoribosyltransferase (HPRT). The primers used for the experiments are summarized in Table 1.

**Table 1:** List of primers used for qRT-PCR.

Gene Name	Forward Sequence	Reverse Sequence
**CyclinD1**	5´–CGTGGCCTCTAAGATGAAGG –3´	5´–CCACTTGAGCTTGTTCACCA –3´
**AREG**	5´–CTGCTGGTCTTAGGCTCAGG –3´	5´–TTTCGCTTATGGTGGAAACC –3´
**Cyr61**	5´–TGCTGTAAGGTCTGCGCTAA –3´	5´–AGGGTCTGCCTTCTGACTGA –3´
**CTGF**	5´–CCACCCGAGTTACCAATGAC –3´	5´–GACAGGCTTGGCGATTTTAG –3´
**ANLN**	5´–TCAATAGCAGCAGTGTTCAGC –3´	5´–GATTTTGTGCCTCACGGTTT –3´
**Diaph3**	5´–TAATGGGCTACTATGCTGTCG –3´	5´–CTCTTTCTCTGCTCGCTCTTT –3´
**Diaph1**	5´–CCCTTTGGATTTGGGGTTC –3´	5´–AGCGGTCCTCCTTCACCTT –3´
**RhoA**	5´–GAAGTCAAGCATTTCTGTCCA –3´	5´–CTCACTCCATCTTTGGTCTTTG –3´
**Rock1**	5´–TTCTGGGAAGAAAGGGACATC –3´	5´–AGGCACGTCATAGTTGCTCAT –3´
**Rac1**	5´–TGTCCCAATACTCCTATCATCC –3´	5´–TACAACAGCAGGCATTTTCTC –3´
**CDC42**	5´–AGTGTGTTGTTGTTGGTGATG –3´	5´–GAGTCTTTGGACAGTGGTGAG –3´
**MyoD**	5´–CTACCCAAGGTGGAGATCCTG –3´	5´–CACTGTAGTAGGCGGTGTCGT –3´
**YAP**	5´–AAGGAGAGACTGCGGTTGAA –3´	5´–CCTGAGACATCCCAGGAGAA –3´
**HPRT**	5´–TGTTGTTGGATATGCCCTTG –3´	5´–ACTGGCAACATCAACAGGACT –3´

### Fluorescence staining

C2C12 cells were seeded either at a density of 9*10^4^ or 2*10^3^ cells/well on 0.2 mm-thick Thermanox plastic slides (Thermo Scientific, Rochester, NY, USA). The previously described cultivation, starvation, and stimulation procedures were followed. Two hours after the FLIPUS-treatment, the cells were fixed with 4% paraformaldehyde. The slides were washed with PBS and incubated for 1 h at room temperature in Phalloidin-Tetramethylrhodamine B isothiocyanate (Sigma Aldrich, Steinheim, Germany), diluted at 1:40 ratio in PBS. The slides were rinsed with PBS and incubated for 20 min in 5 μM Hoechst 33342 solution. Finally, after washing the slides twice with PBS, they were mounted in Flouromount-G^®^ on glass objective slides covered with glass slips and imaged with Leica TCS SP5II confocal microscope. Images were taken at 5 different locations on each slide. At each location, 7 μm Z-stacks were taken with a step size of 1 μm. Imaging settings, including laser and detector- parameters, were kept the same for both stimulated and unstimulated samples. The images were processed by Fiji Software: an average Phalloidin intensity of Z-projections was measured and normalized to the corresponding cell number (plugin of Dr. Kurt De Vos from the University of Sheffield). For unstimulated and stimulated groups 5233 and 5189 cells were counted, respectively. The sparsely seeded cells were only stained for exemplary purposes.

### Statistical analysis

Significance of differences in the evaluated parameters was assessed by one-way ANOVA followed by post-hoc multi-comparison Tukey-Kramer tests using the MATLAB statistics toolbox (Matlab 2015b, The MathWorks, Natick, MA, USA). All data were expressed as mean ± standard deviation (SD). The differences were considered to be significant, if *p*-value was smaller than 0.05.

## Results

### FLIPUS enhances cell proliferation in a YAP dependent manner

**Fig 1:**
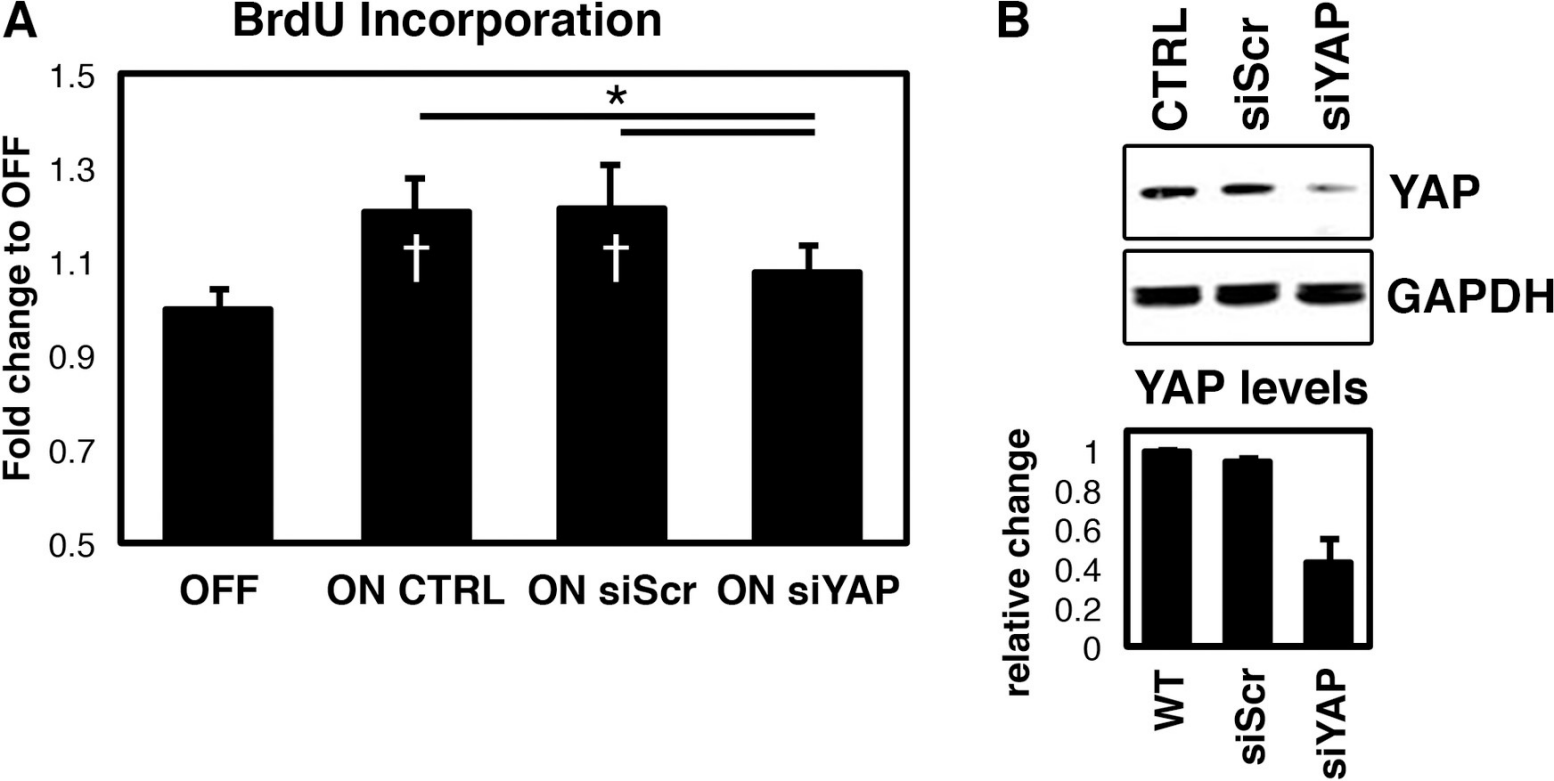
FLIPUS enhances cell proliferation in a YAP-dependent manner. **A:** BrdU incorporation in non-sonicated (OFF), FLIPUS-treated (ON CTRL), FLIPUS-treated scrambled siRNA transfected (ON siScr) and YAP-targeting siRNA transfected (ON siYAP) C2C12 cells. Each experiment was repeated four times in duplicate. **B:** Representative picture of western blot analysis confirming efficient YAP knockdown on protein levels with near-infrared fluorescent intensity quantification of four biological replicates Data are presented as mean ± SD *p < 0.05 indicate changes between the sonicated groups ^†^*p* < 0.05 indicate changes between FLIPUS-treated and non-sonicated cells for a selected treatment.

To characterize the nature of the pro-regenerative effects of FLIPUS observed in clinical applications, we analyzed its effect on proliferation of mesenchymal progenitors. BrdU-ELISA assays revealed that FLIPUS enhanced the proliferation of C2C12 cells (A). Due to our recent findings that activation of TEAD binding sequences is enhanced after 5-min-long FLIPUS exposure [19] and that Yes-associated Protein (YAP) is one of the major transcriptional co-factors of TEADs, we explored whether the observed increase in proliferation is mediated by YAP. Indeed, YAP knockdown (siYAP) significantly reduced the cell growth induced by ultrasound stimulation (Fig 1A, S1 Table). siYAP efficiency was confirmed by western blotting (Fig 1B).

Although a subtle increase (^~^ 21%) in cellular proliferation after the ultrasound treatment was observed, these results are considerable, because only 0.64% of densely seeded cells were capable of undergoing mitosis after the starvation (S1 Fig). Moreover, full media conditions containing FCS, a cocktail of various growth factors, increased the number of cell divisions only up to 1.09%, corresponding to 70% raise (S1 Fig).

### FLIPUS reduces phosphorylation of YAP at Serine 127

**Fig 2:**
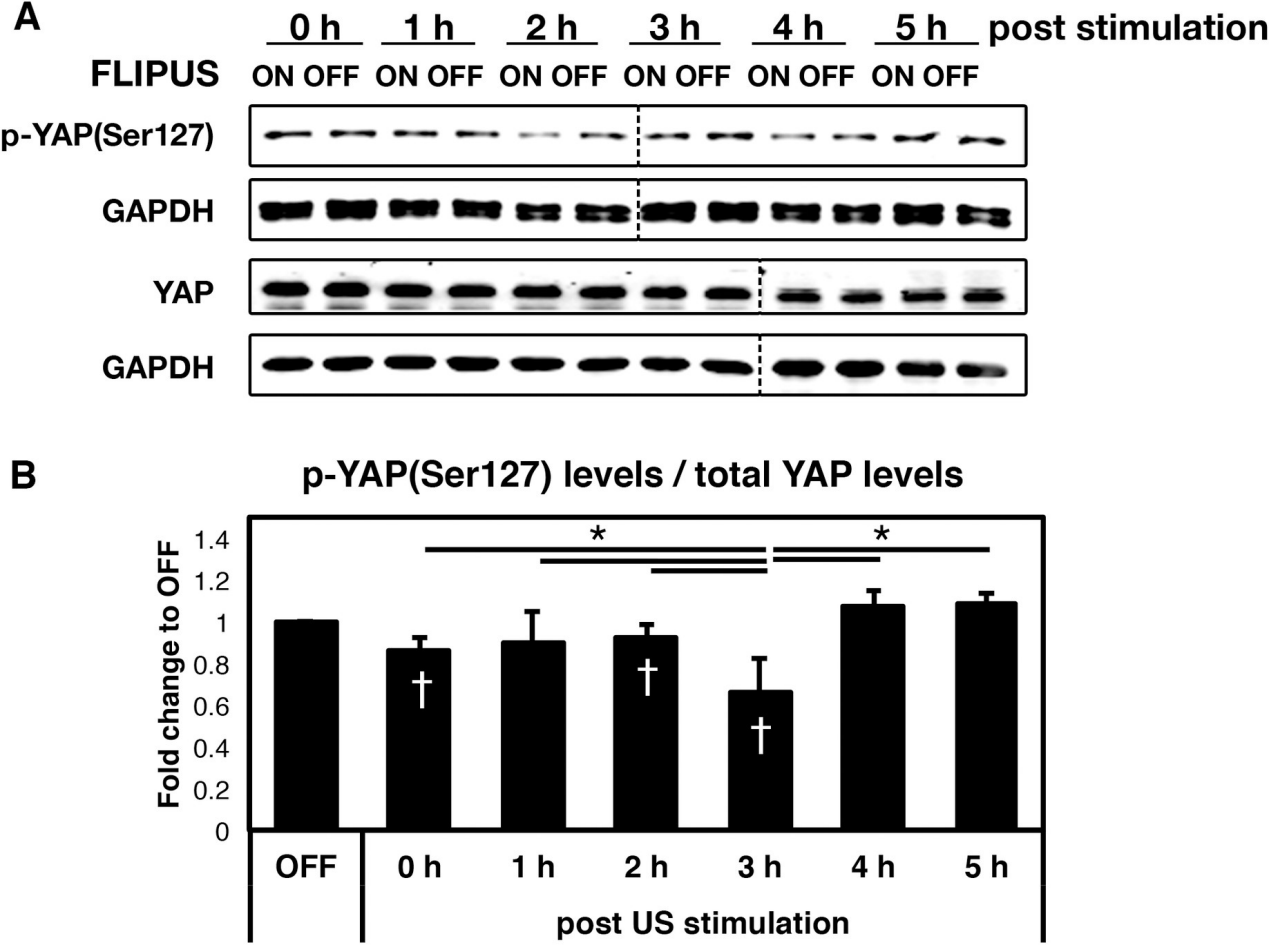
FLIPUS reduces phosphorylation of YAP at Serine 127. **A:** Representative picture of western blotting analysis of C2C12 cells harvested 0 h, 1 h, 2 h, 3 h, 4 h, and 5 h after 5-min FLIPUS stimulation. **B:** Near-infrared fluorescent intensity quantification of p-YAP(Ser127) levels normalized to total YAP levels of at least three biological replicates per time point presented as mean fold change of FLIPUS-treated cells compared to non-sonicated controls (OFF) of the corresponding time point ± SD *p < 0.05 indicate changes between the time points, ^†^*p* < 0.05 indicate changes between FLIPUS-treated and untreated cells for a selected time point. The vertical dashed line represents location of the protein molecular weight marker, which was not included in the image.

Since FLIPUS increases cell proliferation in a YAP dependent manner, we next wanted to dissect the role of YAP in the cellular response to ultrasound stimulation. The subcellular localization of YAP and therefore its transcriptional activity are regulated by phosphorylation. Time resolved analyses of YAP phosphorylation at Serine 127 after 5 min of FLIPUS stimulation revealed a reduction of p-YAP(Ser127) levels after 2 h, reaching a minimum at 3 h and returning to basal level 4 h after the FLIPUS exposure, whereas total YAP levels remained unchanged (Fig 2A and 2B, S2 Fig, S2 Table). There was also a subtle, but statistically significant decrease in p-YAP(Ser127) observed right after the stimulation.

### FLIPUS promotes YAP nuclear accumulation

**Fig 3:**
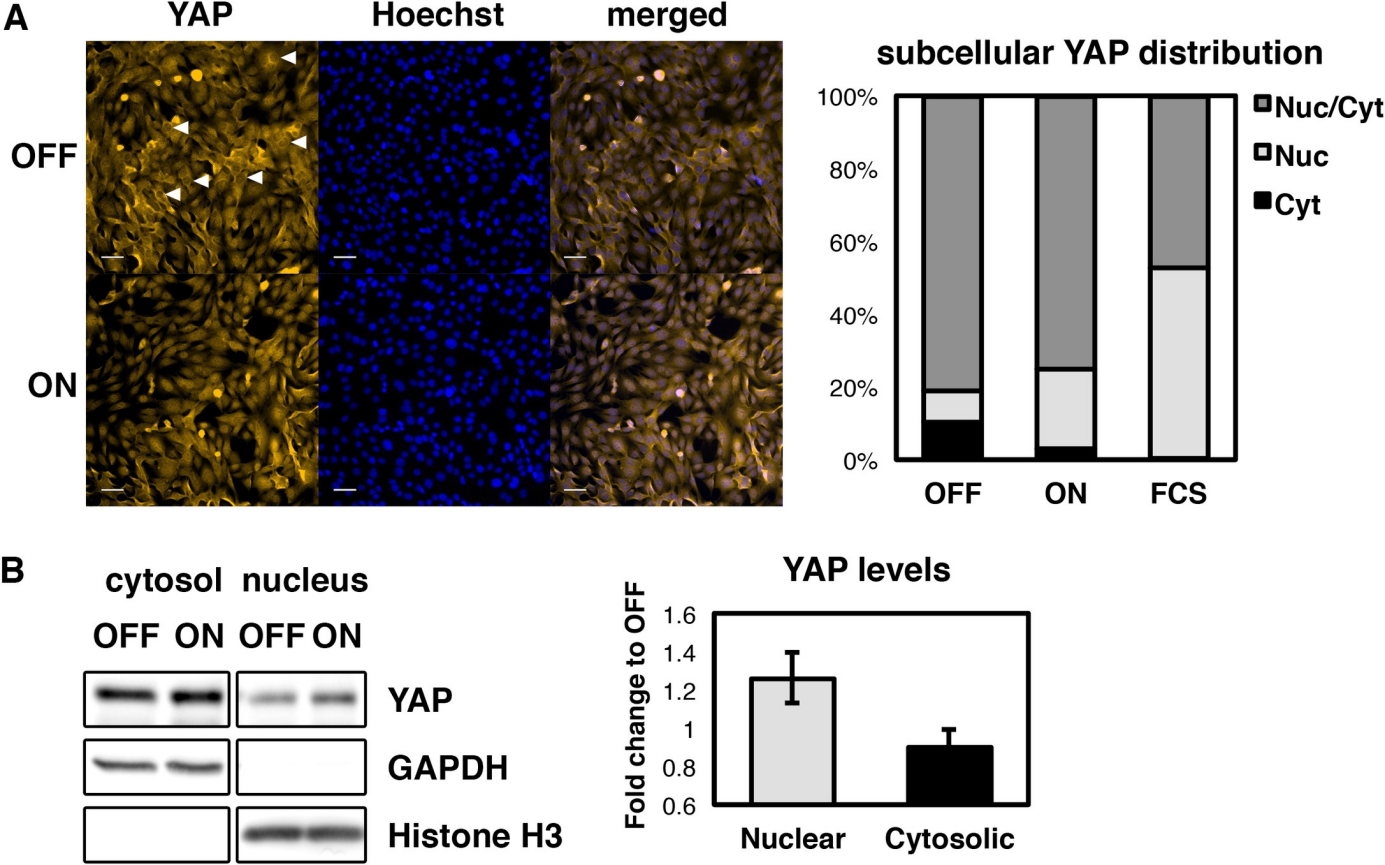
FLIPUS promotes nuclear accumulation of YAP. **A:** Representative immunofluorescence images of YAP localization in non-sonicated (OFF) and FLIPUS-treated (ON) C2C12 cells, fixed 2 hours after stimulation. Scale bar size is 100 μm. Arrows indicate cells with predominantly cytoplasmic YAP. Images were quantified by classification of cells into mostly cytosolic YAP (Cyt), mostly nuclear YAP (Nuc), or equal distribution (Nuc/Cyt) from three biological replicates. **B:** Representative picture of western blot analysis of cell fractionation assays harvested 2 hours after stimulation with quantification of three biological replicates. Data are presented as mean ± SD.

Phosphorylation of YAP at Serine 127 leads to its sequestration in the cytoplasm and therefore dictates its subcellular localization. Since FLIPUS leads to a decrease in p-YAP(Ser127) levels, we performed immunofluorescence stainings to analyze the impact of ultrasound stimulation on subcellular localization of YAP. FLIPUS stimulation resulted in an increased number of cells with primarily nuclear YAP localization after 2 hours compared to unstimulated cells, whereas the number of cells with predominantly cytoplasmic YAP decreased in response to FLIPUS (Fig 3A, S3 Table). Confocal imaging of the slides confirms this finding, showing more intense YAP staining in the cells nuclei after the FLIPUS-stimulation (S3 Fig). Stimulation with fetal calf serum (FCS), which is known to result in YAP dephosphorylation and nuclear translocation [20], was used as a positive control. Results of cell fractionation assays corroborate the findings of our immunofluorescence stainings (Fig 3B, S4 Table), revealing an increase in nuclear and decrease in cytosolic YAP levels 2 h after the stimulation. FLIPUS thus induces YAP translocation to the nucleus.

### Gene expression of YAP targets is changed in response to FLIPUS

Together with transcription factors such as TEADs, nuclear YAP drives the expression of different target genes, some of which are associated with cytokinesis and cell cycle progression. Since FLIPUS leads to an accumulation of YAP in the nucleus (Fig 3), increased activation of TEAD binding sites [19], and enhanced YAP dependent cellular proliferation (Fig 1), we sought to analyze the impact of FLIPUS on the expression of YAP target genes. We established that cell-cycle-related genes AREG, CyclinD1, and Cyr61, together with cytoskeletal stabilizing genes ANLN, Diaph1, and Diaph3, are YAP target genes, since siRNA mediated knockdown of YAP (siYAP) resulted in a significant decrease of their expression (S4 Fig, S5 Table). Furthermore, expression of CTGF and myogenic transcription factor MyoD were upregulated by YAP silencing in C2C12 cells (S4 Fig, S5 Table).

**Fig 4:**
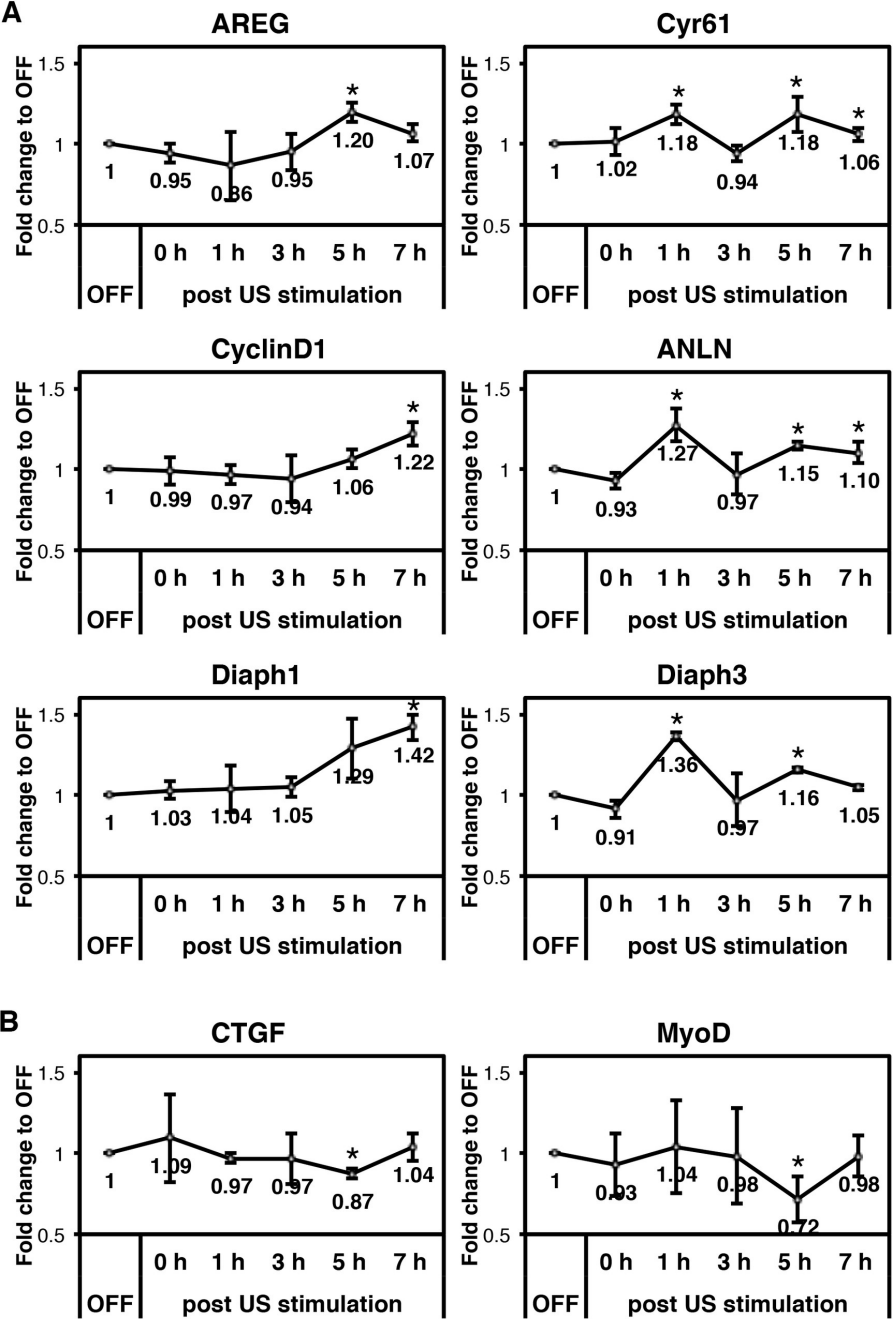
FLIPUS regulates gene expression of YAP targets. Fold change in the mean normalized mRNA expression of genes in C2C12 cells harvested 0 h, 1 h, 2 h, 3 h, 5 h, and 7 h after 5-min-long FLIPUS stimulation compared to non-sonicated controls (OFF) of the corresponding time point. Results from three biological replicates are presented as mean ± SD. *p < 0.05 indicate changes between FLIPUS-treated and untreated cells for a selected time point. **A:** Genes that are downregulated by YAP knockdown and upregulated by FLIPUS. **B:** Genes that are upregulated by YAP knockdown and downregulated by FLIPUS.

Next, we analyzed changes of YAP target genes in response to FLIPUS. Genes that were downregulated by YAP silencing showed increased expression after ultrasound stimulation, reaching significance 5 to 7 hours after the sonication (Fig 4A, S6 Table). Conversely, expression of MyoD and CTGF–genes that were upregulated after siYAP–was decreased 5 h after FLIPUS treatment (Fig 4B, S6 Table). ANLN, Diaph3, and Cyr61 mRNA levels exhibited a cyclic regulation, showing an early increase 1 h after sonication as well (Fig 4A, S6 Table).

### FLIPUS induces expression of actin stabilizing genes and promotes actin bundling

**Fig 5:**
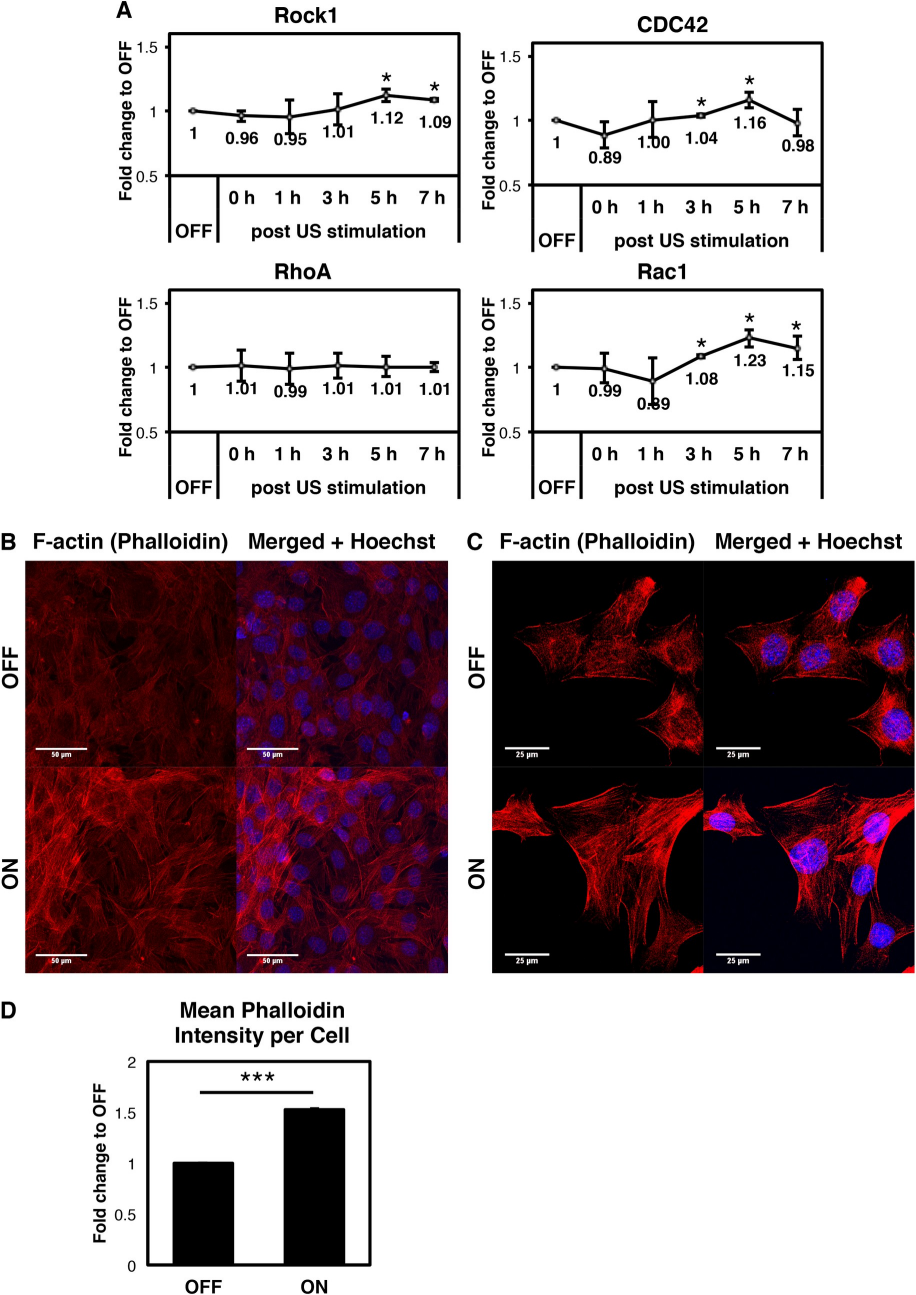
FLIPUS affects cellular cytoskeleton. **A:** Fold change in the mean normalized mRNA expression of genes in C2C12 cells harvested 0 h, 1 h, 2 h, 3 h, 5 h, and 7 h after 5-min-long FLIPUS stimulation compared to non-sonicated controls (OFF) of the corresponding time point. Results from three biological replicates are presented as mean ± SD. *p < 0.05 indicate changes between FLIPUS-treated and untreated cells for a selected time point. **B:** Exemplary F-actin stainings with Phalloidin in densely seeded non-sonicated (OFF) and FLIPUS-treated (ON) C2C12 cells. **C:** Exemplary F-actin stainings with Phalloidin in sparsely seeded non-sonicated (OFF) and FLIPUS-treated (ON) C2C12 cells. **D:** Summary of quantification results of average Phalloidin intensity normalized to cell number per image. Results from three biological replicates are presented as mean ± SD. ***p < 0.01 indicate changes between FLIPUS-treated and untreated cells.

We next asked how the cells transduce mechanical stimulation by FLIPUS into a biological response, leading to an increased YAP activity. Interestingly, some of the ultrasound responsive YAP target genes we analyzed, namely ANLN, Diaph1, and Diaph3, play a role in stabilization of the actin cytoskeleton (Fig 4A). Additionally, we examined the expression of the small GTPases RhoA, and its effector Rock1, CDC42, and Rac1, which did not show changes in expression after siYAP (S4 Fig). Rock1, CDC42, and Rac1 mRNA levels significantly increased 3 to 5 h after FLIPUS stimulation, partially persisting for 7 hours (Fig 5A, S6 Table). RhoA expression did not change after sonication.

These responses led us to hypothesize that the cells react to mechanical ultrasound stimulation by adapting their actin cytoskeleton. We, therefore, performed F-actin stainings to visualize potential changes in the actin cytoskeleton in response to FLIPUS. Confocal imaging of Phalloidin stained FLIPUS-treated cells at 40 x magnification revealed higher fluorescence intensity per ultrasound-treated cell (Fig 5B and 5D). In order to observe actin architecture in detail, images of sparsely seeded cells at 63 x magnification were taken. The actin cytoskeleton of unstimulated cells appeared weak and dispersed (Fig 5C), whereas FLIPUS-treated cells formed thick F-actin bundles. This is in agreement with Dupont *et al*. [3], who showed that increased cell spreading and tension of the actomyosin cytoskeleton lead to activation of YAP.

## Discussion

The process of tissue regeneration heavily relies on the proliferative potential of cells, required to reconstruct the impaired site [4]. Inadequate cellular functioning eventually leads to tissue deterioration and significant organ disabilities. The transcriptional coactivator YAP, an effector of the mechanosensitive Hippo pathway, regulates cellular proliferative potential, which in turn decides the outcome of successful tissue regeneration [21]. Our recent findings suggest that Focused Low-Intensity Pulsed Ultrasound (FLIPUS) mechanical stimulation can increase YAP activity, resulting in enhanced cellular proliferation of C2C12 mesenchymal progenitor cells. Silencing of YAP abolishes this pro-proliferative effect of FLIPUS.

Inspired by our previous results showing increased activation of TEAD binding sites in response to 5-min-long FLIPUS exposure in C2C12 cells [19], we investigated if the mechanotransducer YAP, a major coactivator of TEADs, mediates cellular responses to ultrasound exposure. We found that FLIPUS mechanical stimulation, indeed, reduced phosphorylation of YAP at Serine 127, which concomitantly results in an enhanced nuclear localization of YAP, as demonstrated by immunofluorescent stainings. These results were additionally confirmed by fractionation assays, revealing higher nuclear YAP levels after the sonication. Phosphorylation at Serine 127 leads to the retention of YAP in the cytosol by binding to 14-3-3 proteins [7]. Since FLIPUS reduces p-YAP(Ser127) levels, more unphosphorylated YAP can thus accumulate in the nucleus to activate target gene transcription after sonication.

The critical role of YAP in defining the cellular commitment of C2C12 cells has been demonstrated by Watt *et al*. [22]. Their study emphasized that overexpression of the phosphorylation-deficient YAP(S127A) mutant, which exhibits increased transcriptional activity by avoiding cytoplasmic retention, delayed myogenic differentiation of C2C12 cells and promoted their proliferation. Judson *et al*. [23] supported this finding by showing that YAP expression was elevated during activation of primary murine satellite cells, while it was reduced when the cells underwent differentiation. These reports suggest that phosphorylation and therefore inactivation of YAP is crucial for cell cycle exit and muscle maturation. In the study by Watt *et al*. [22], the overexpression of YAP1 S127A mutant was achieved via its exogenous introduction, while in our experimental set-up only endogenous transient phosphorylated vs unphosphorylated YAP changes were evaluated. Although it is tricky to compare the studies directly, some conclusions could be drawn from the evaluation of the data: the overexpression of YAP1 S127A in C2C12s resulted in approximately 35% increase in CyclinD1 expression, whereas expression of myogenesis-promoting transcription factor Mef2c was decreased by around 40%, in comparison to wild type YAP1- transfected cells. In our experimental work, we observed about 20% increase in CyclinD1 and 30% decrease in MyoD1 expression after the FLIPUS-treatment. However, it is important to note that different time points were analyzed in the studies.

To verify the observations made by Watt *et al*., we selected several genes known to be regulated by YAP and investigated whether their expression is affected by YAP knockdown in our cells. AREG, Cyr61, and CyclinD1 have been numerously demonstrated to be YAP target genes with cell cycle promoting properties [24]. The YAP-mediated transcriptional regulation of ANLN, Diaph1 and Diaph3 has been previously shown by Calvo *et al*. [25]. The authors suggested that these proteins contribute to the extracellular matrix (ECM) stiffening, promoting YAP nuclear activity in a feed-forward loop. Diaph1, an effector of RhoA, is an actin nucleating formin, which regulates the elongation of actin filaments towards their barbed ends [26]. ANLN protein regulates bundling of F-actin and is also enriched and co-localized with myosin II in the cleavage furrow during cytokinesis [27]. ANLN binding to formin Diaph3 stabilizes its conformation crucial for cell division [28]. Here, we were able to confirm that the pro-proliferative genes AREG, Cyr61, CyclinD1, as well as the cytoskeleton stabilizing and cytokinesis-associated genes ANLN, Diaph1, and Diaph3 are downregulated when YAP is knocked down in C2C12 cells. The expression of other cytoskeletal genes, such as small GTPases RhoA, CDC42, and Rac1, as well as RhoA’s effector Rock1 kinase, were not affected by YAP silencing. On the contrary, the expression of the myogenic transcription factor MyoD was enhanced after YAP knockdown. These results are in line with those of Watt *et al*. [22], who identified YAP as a negative regulator of myogenesis in C2C12.

We next analyzed changes in expression of the above-mentioned genes after FLIPUS stimulation. The six YAP target genes, AREG, Cyr61, CyclinD1, ANLN, Diaph1, and Diaph3 were found to be enhanced 5 h to 7 h after 5-min-long sonication. The Cyr61, ANLN and Diaph3 gene expression was enhanced in cyclic manner, with the first burst 1 h after the ultrasound treatment. YAP transcriptional co-activator could have also mediated this event, since there is a decrease in p-YAP (Ser127) levels right after the FLIPUS exposure (Fig 2A and 2B). Stimulation with FLIPUS reduced expression of MyoD 5 h after ultrasound exposure. This suggests that FLIPUS treatment induces an early YAP-mediated transcriptional response supporting stress fiber formation, cytokinesis and proliferation of C2C12 cells, while delaying cellular specification.

Strikingly, the expression of CTGF, which has been previously shown to be enhanced via YAP activation [5, 8, 10], increased by YAP silencing and decreased in response to FLIPUS stimulation. Our observations could be supported by Hishikawa *et al*. [29], who found that CTGF suppresses cellular viability and induces apoptosis in smooth muscle cells *via* activation of caspase 3 cascade. However, further experimental work is required in order to establish the complete underlying mechanism.

Interestingly, the expression of Rock1, CDC42, and Rac1 mRNA was also upregulated after FLIPUS treatment. Previous studies have shown that enhanced activity of CDC42, Rac1 [30], and Rock1 [31] attenuates myogenic differentiation, suggesting that FLIPUS could inhibit myogenesis through an additional mechanism, which is not, or only indirectly, mediated by YAP.

We further propose that the molecular mechanism linking FLIPUS and YAP activation is mediated by the actin cytoskeleton, as transcription of genes stabilizing actin fibers is regulated in response to ultrasound (Fig 5A). The dependence of YAP activity on cytoskeletal tension was first demonstrated by original work of the group of Stefano Piccolo [24]. A number of studies show that actin fibers relay information about the cell shape, substrate properties, and mechanical stimulation to the Hippo pathway and YAP activity. Reddy *et al*. [13] found that drug-induced actin polymerization resulted in a decrease of YAP phosphorylation at Ser127 and an increased nuclear localization of YAP. A study by Zhong *et al*. [32] demonstrated that rat MSCs and chondrocytes subjected to the gradual increase of shear-stress, which was generated by a microfluidic bioreactor, led to more intensive YAP nuclear localization through the formation of stress-fibers. This in turn enhanced the proliferative potential of the cells. A similar principle is likely the base of FLIPUS mediated YAP activation.

Imaging of cytoskeleton in sonicated cells at high magnification (Fig 5B and 5C) revealed thick bundling of actin fibers, which, most likely, signifies stabilization of the cytoskeleton in line with increased expression of Diaph1, Diaph3 and ANLN. The unstimulated cells also developed stress fibers, which is anticipated on a stiff polystyrene plastic, however, they appeared fuzzy and dispersed (Fig 5B and 5C). The observed bundling of stress fibers could also stem from enhanced formation of the, so-called, actin cap. The actin cap is a perinuclear organelle, consisting of thick and parallel actin bundles attached to the nuclear envelope [33]. These filaments are crucial for mechanotransduction and serve as a link transmitting the mechanosignal into the nucleus [34]. Indeed, YAP-signaling was previously linked to the actin cap in cells [35]. However, this hypothesis needs further investigation and other cortical actin structures could also be involved in the mechanism.

As a next step, we will shift further investigation of the YAP-mediated mechanism to 3D cultures in scaffolds. The substrate stiffness then can be varied, mimicking natural tissue architecture, and changes in FLIPUS-induced cellular mechanoresponse can be evaluated, revealing ultrasound-mediated regenerative mechanisms without constrain to polystyrene plastic. Most certainly, further investigation of the events upstream of YAP activation, such as the involvement of the evolutionary conserved Hippo kinase cassette, is necessary to expand our knowledge of FLIPUS-triggered biological and physical responses.

**Fig 6:**
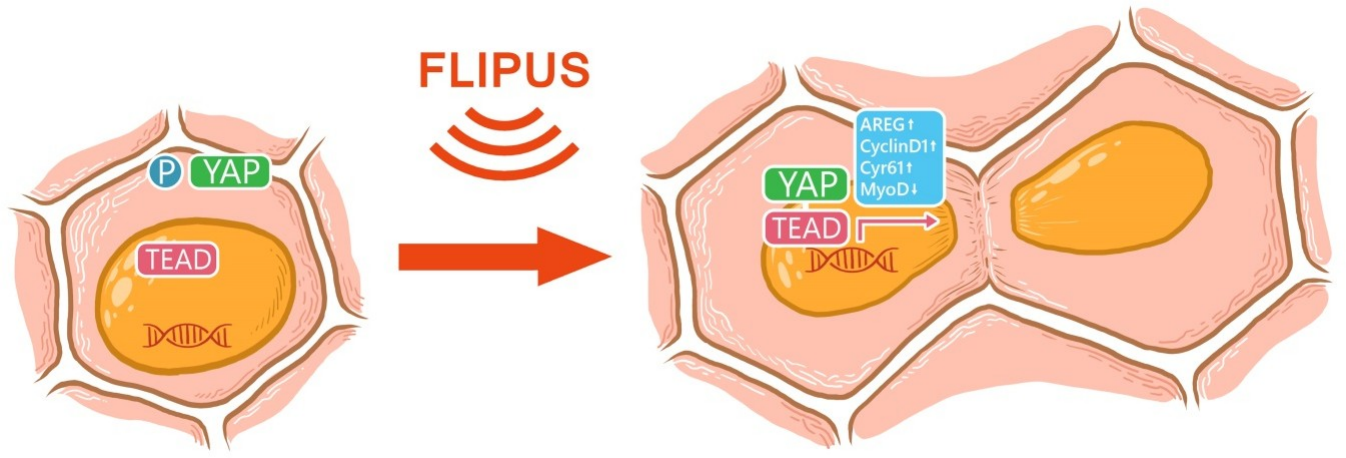
Hypothesized mechanotransduction mechanism regulating the pro-proliferative response of C2C12 cells to FLIPUS. FLIPUS treatment leads to dephosphorylation and nuclear translocation of YAP, resulting in the increased transcription of pro-proliferative genes.

Taken together, our results suggest that FLIPUS reduces phosphorylation of YAP at Ser127, through stabilization of actin cytoskeleton. This activates YAP, leading to its nuclear translocation, where pro-proliferative, cytokinesis-related, and anti-differentiation changes in expression occur, which in turn drives division of C2C12 cells (Fig 6).

## Supporting information

S1 Fig Number of C2C12 cells undergoing mitosis. **A:** Exemplary images of C2C12 cells stained for phospho-histone H3 (Ser28) in starving (0% FCS DMEM) and full (10% FCS DMEM) media conditions. Scale bar is 50 μm. **B:** Quantification results of cells undergoing mitosis normalized to total cell number. Results are from three biological replicates presented as mean ± SD, **p < 0.01.
(TIF)

S2 Fig FLIPUS does not change total YAP protein levels. Near-infrared fluorescent intensity quantification of YAP protein levels normalized to GAPDH levels of at least three biological replicates per time point, presented as mean fold change of FLIPUS-treated cells compared to non-sonicated controls (OFF) of the corresponding time point ± SD.
(TIF)

S3 Fig Exemplary images of YAP localization in response to FLIPUS taken by confocal microscopy. Scale bar size is 25 μm.
(JPG)

S4 Fig Validation of YAP target genes. Fold change in the mean normalized mRNA expression of genes in C2C12 cells transfected with siRNA targeting YAP (siYAP) compared to cells transfected with scrambled siRNA as a control (siScr). Results from three biological replicates are presented as mean ± SD, *p < 0.05, **p < 0.01, and ***p < 0.001.
(TIF)

S1 Table FLIPUS enhances cell proliferation in a YAP-dependent manner. Proliferation of untransfected (CTRL), siScr- (ON siScr) and siYAP-transfected (ON siYAP) C2C12s after FLIPUS stimulation. Values are normalized to unstimulated controls.
(DOCX)

S2 Table FLIPUS reduces phosphorylation of YAP at Serine 127. Quantification of p-YAP(Ser127) amounts normalized to total YAP amounts. GAPDH was used as a loading control. The values for each time point represent fold induction over unstimulated control.
(DOCX)

S3 Table FLIPUS promotes nuclear accumulation of YAP. Quantification of YAP-localization: cells with YAP-filled nuclei (Nuc), cytosol-localized YAP (Cyt) and nucleus-cytosol distributed YAP after FLIPUS stimulation. Each value is normalized to unstimulated control.
(DOCX)

S4 Table Densitometric quantification of YAP fractionation assay. YAP content in nucleus (Nuc) and cytosol (Cyt) after FLIPUS stimulation. Each value is normalized to unstimulated control.
(DOCX)

S5 Table Validation of YAP target genes. Relative mRNA expression in siYAP C2C12 cells, normalized to expression level in siScr–transfected cells.
(DOCX)

S6 Table Summary of mRNA expression levels after FLIPUS-stimulation of C2C12s. Each stimulated value is normalized to its own unstimulated control for every time point.
(DOCX)
