# Supplementary figures and images for: Functional regulation of YAP mechanosensitive transcriptional coactivator by Focused Low-Intensity Pulsed Ultrasound (FLIPUS) enhances proliferation of murine mesenchymal precursors
Source: PLoS One. 2018 Oct 26;13(10):e0206041. doi: 10.1371/journal.pone.0206041 (PMC6203358; doi:10.1371/journal.pone.0206041)

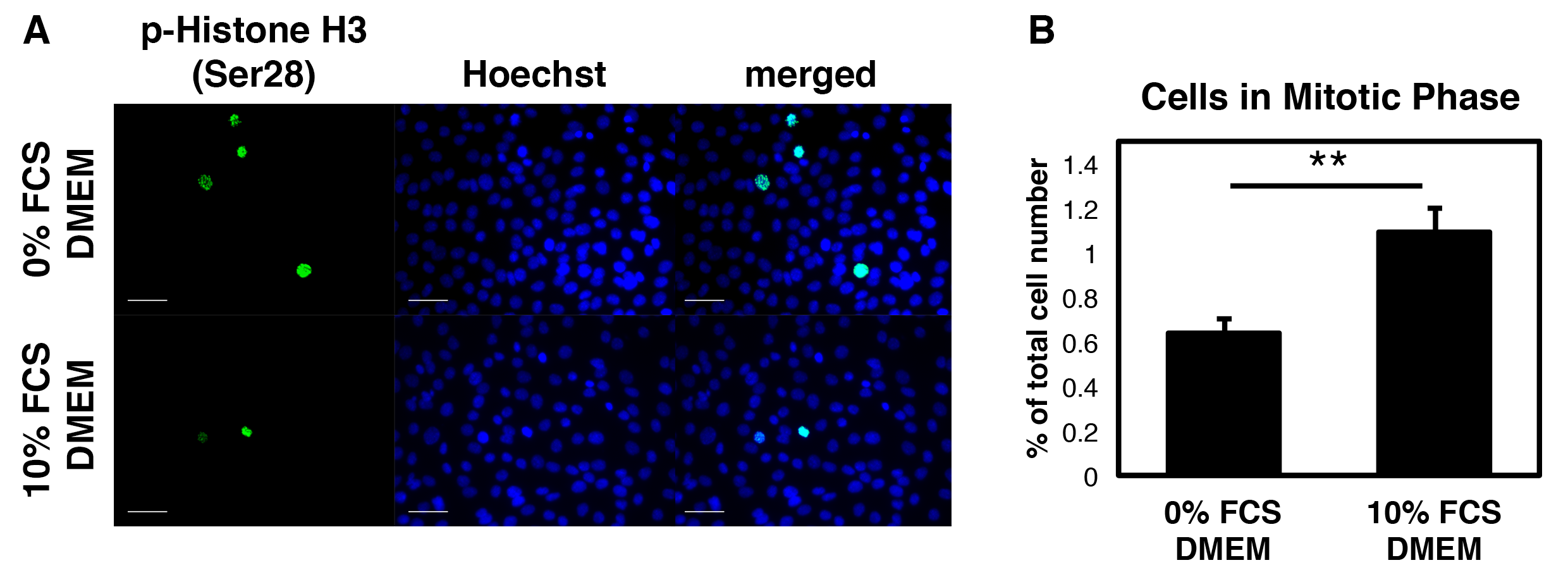

Supplement: S1 Fig — A: Exemplary images of C2C12 cells stained for phospho-histone H3 (Ser28) in starving (0% FCS DMEM) and full (10% FCS DMEM) media conditions. Scale bar is 50 μm. B: Quantification results of cells undergoing mitosis normalized to total cell number. Results are from three biological replicates presented as mean ± SD, **p < 0.01. (TIF) [file pone.0206041.s001.tif]

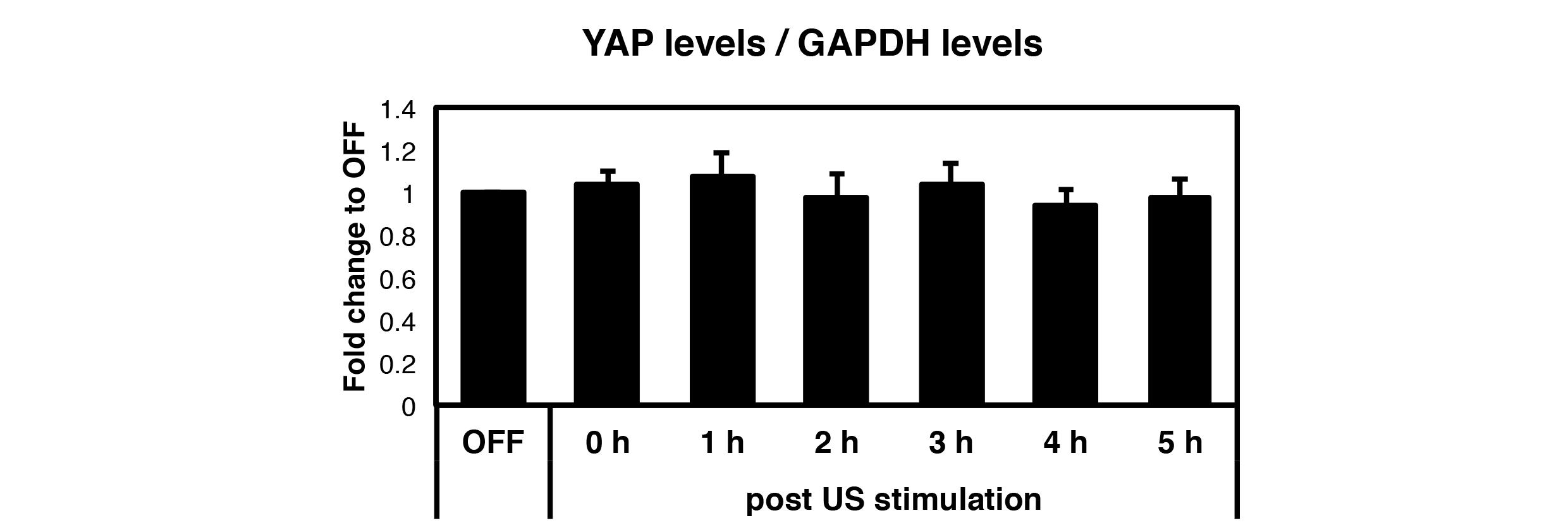

Supplement: S2 Fig — Near-infrared fluorescent intensity quantification of YAP protein levels normalized to GAPDH levels of at least three biological replicates per time point, presented as mean fold change of FLIPUS-treated cells compared to non-sonicated controls (OFF) of the corresponding time point ± SD. (TIF) [file pone.0206041.s002.tif]

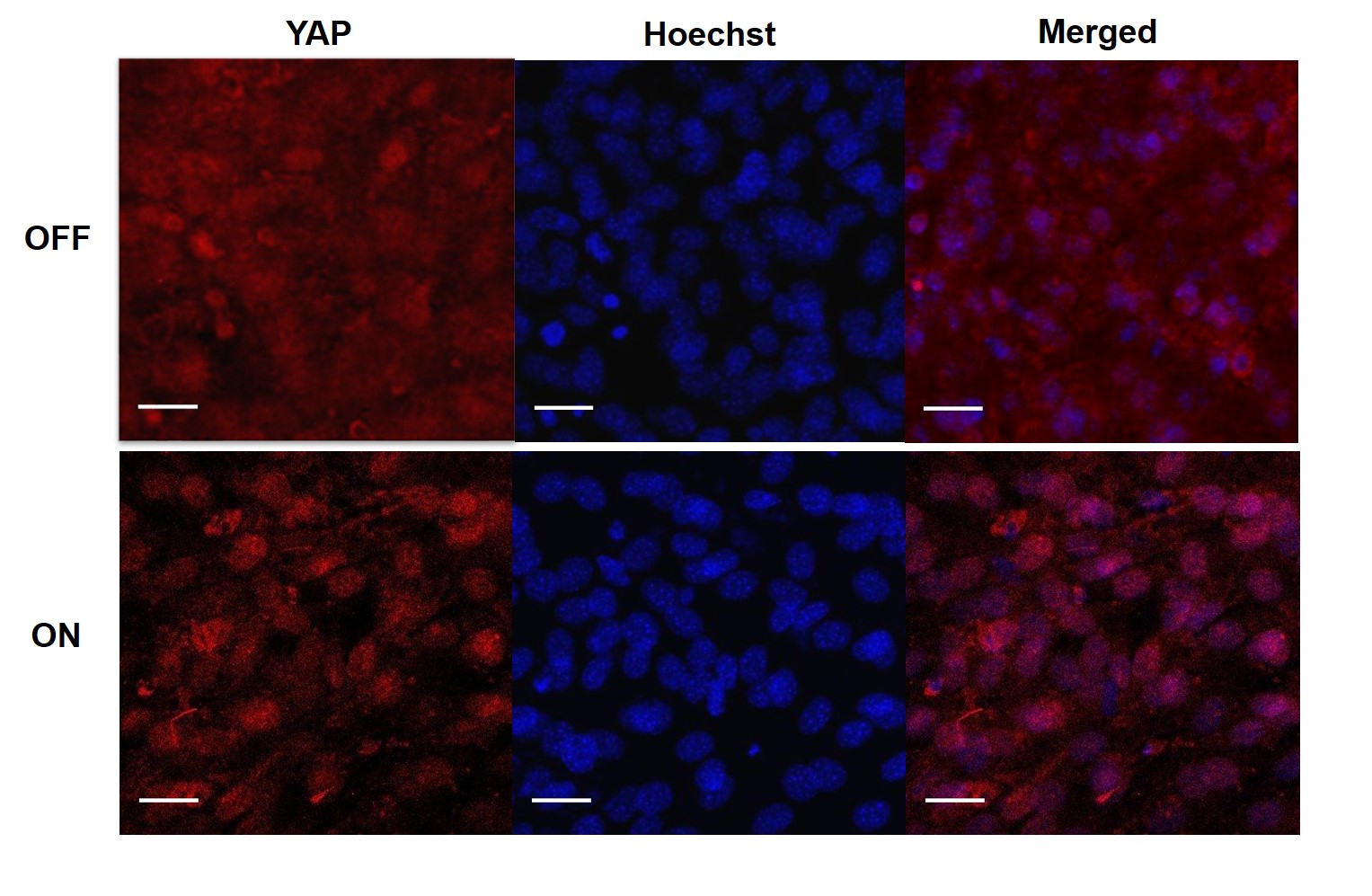

Supplement: S3 Fig — Scale bar size is 25 μm. (JPG) [file pone.0206041.s003.jpg]

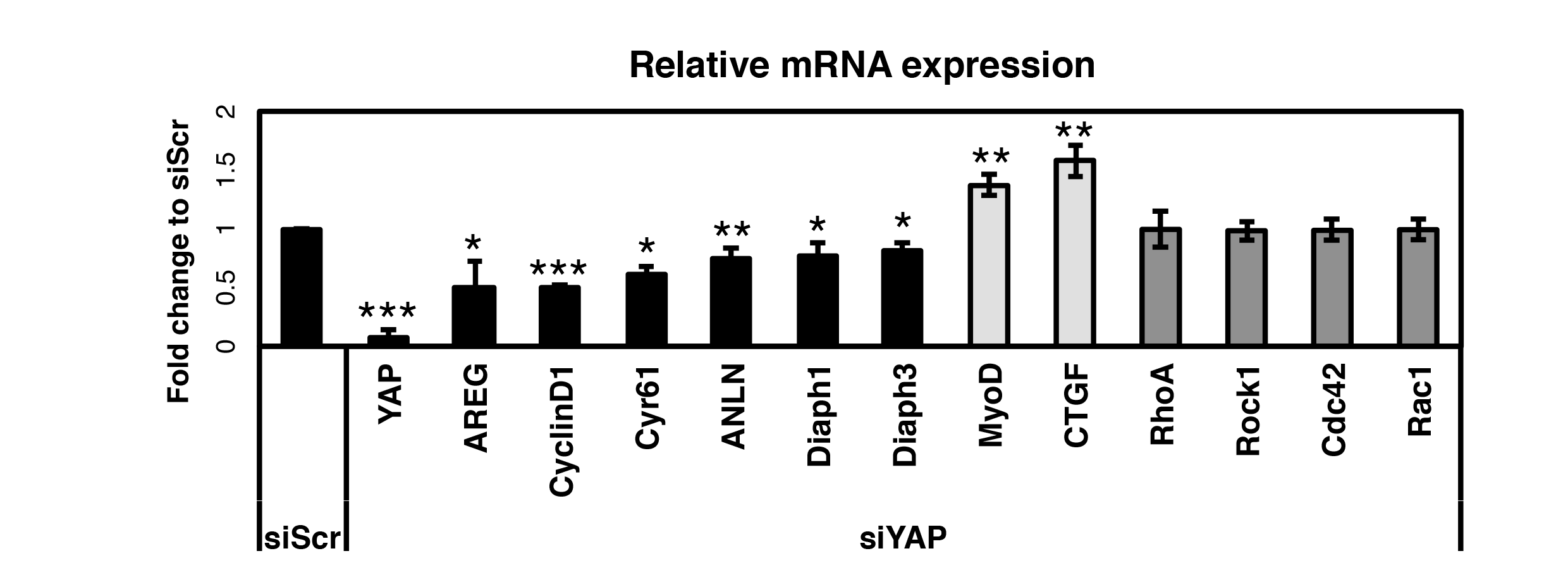

Supplement: S4 Fig — Fold change in the mean normalized mRNA expression of genes in C2C12 cells transfected with siRNA targeting YAP (siYAP) compared to cells transfected with scrambled siRNA as a control (siScr). Results from three biological replicates are presented as mean ± SD, *p < 0.05, **p < 0.01, and ***p < 0.001. (TIF) [file pone.0206041.s004.tif]
