# Supplementary material for: Functional regulation of YAP mechanosensitive transcriptional coactivator by Focused Low-Intensity Pulsed Ultrasound (FLIPUS) enhances proliferation of murine mesenchymal precursors
Source: PLoS One. 2018 Oct 26;13(10):e0206041. doi: 10.1371/journal.pone.0206041 (PMC6203358; doi:10.1371/journal.pone.0206041)
Supplement: S1 Table — Proliferation of untransfected (CTRL), siScr- (ON siScr) and siYAP-transfected (ON siYAP) C2C12s after FLIPUS stimulation. Values are normalized to unstimulated controls. (DOCX) [file pone.0206041.s005.docx]

|  | **Mean** | **SD** | ***p*-value** |
| --- | --- | --- | --- |
| **ON CTRL** | 1.208 | 0.067 | 4.04E-06 |
| **ON siScr** | 1.211 | 0.094 |  |
| **ON siYAP** | 1.075 | 0.060 | n.s. |
