# Supplementary material for: Functional regulation of YAP mechanosensitive transcriptional coactivator by Focused Low-Intensity Pulsed Ultrasound (FLIPUS) enhances proliferation of murine mesenchymal precursors
Source: PLoS One. 2018 Oct 26;13(10):e0206041. doi: 10.1371/journal.pone.0206041 (PMC6203358; doi:10.1371/journal.pone.0206041)
Supplement: S2 Table — Quantification of p-YAP(Ser127) amounts normalized to total YAP amounts. GAPDH was used as a loading control. The values for each time point represent fold induction over unstimulated control. (DOCX) [file pone.0206041.s006.docx]

|  | **p-YAP(Ser127) to YAP** | | |
| --- | --- | --- | --- |
|  | **Mean** | **SD** | ***p*-value** |
| **0 h** | 0.86 | 0.06 | 0.007 |
| **1 h** | 0.89 | 0.16 | n.s. |
| **2 h** | 0.93 | 0.05 | 0.0151 |
| **3 h** | 0.66 | 0.16 | 0.0015 |
| **4 h** | 1.08 | 0.07 | n.s. |
| **5 h** | 1.08 | 0.06 | n.s. |
