# Supplementary material for: Functional regulation of YAP mechanosensitive transcriptional coactivator by Focused Low-Intensity Pulsed Ultrasound (FLIPUS) enhances proliferation of murine mesenchymal precursors
Source: PLoS One. 2018 Oct 26;13(10):e0206041. doi: 10.1371/journal.pone.0206041 (PMC6203358; doi:10.1371/journal.pone.0206041)
Supplement: S3 Table — Quantification of YAP-localization: cells with YAP-filled nuclei (Nuc), cytosol-localized YAP (Cyt) and nucleus-cytosol distributed YAP after FLIPUS stimulation. Each value is normalized to unstimulated control. (DOCX) [file pone.0206041.s007.docx]

|  | **Mean** | **SD** | ***p-*Value** |
| --- | --- | --- | --- |
| **Nuc** | 2.44 | 0.45 | 0.0052 |
| **Cyt** | 0.33 | 0.16 | 0.0020 |
| **Nuc/Cyt** | 0.86 | 0.12 | n.s. |
