# Supplementary material for: Functional regulation of YAP mechanosensitive transcriptional coactivator by Focused Low-Intensity Pulsed Ultrasound (FLIPUS) enhances proliferation of murine mesenchymal precursors
Source: PLoS One. 2018 Oct 26;13(10):e0206041. doi: 10.1371/journal.pone.0206041 (PMC6203358; doi:10.1371/journal.pone.0206041)
Supplement: S4 Table — YAP content in nucleus (Nuc) and cytosol (Cyt) after FLIPUS stimulation. Each value is normalized to unstimulated control. (DOCX) [file pone.0206041.s008.docx]

|  | **Mean** | **SD** | ***p*-value** |
| --- | --- | --- | --- |
| **Nuc** | 1.26 | 0.13 | 0.028 |
| **Cyt** | 0.90 | 0.095 | n.s. |
