# Supplementary material for: Functional regulation of YAP mechanosensitive transcriptional coactivator by Focused Low-Intensity Pulsed Ultrasound (FLIPUS) enhances proliferation of murine mesenchymal precursors
Source: PLoS One. 2018 Oct 26;13(10):e0206041. doi: 10.1371/journal.pone.0206041 (PMC6203358; doi:10.1371/journal.pone.0206041)
Supplement: S5 Table — Relative mRNA expression in siYAP C2C12 cells, normalized to expression level in siScr–transfected cells. (DOCX) [file pone.0206041.s009.docx]

| **Gene** | **Mean** | **SD** | ***p*-value** |
| --- | --- | --- | --- |
| **YAP** | 0.08 | 0.06 | 8.4E-06 |
| **AREG** | 0.51 | 0.21 | 0.016 |
| **CyclinD1** | 0.50 | 0.03 | 4.6E-06 |
| **Cyr61** | 0.61 | 0.08 | 0.019 |
| **ANLN** | 0.74 | 0.09 | 0.0081 |
| **Diaph1** | 0.76 | 0.11 | 0.023 |
| **Diaph3** | 0.81 | 0.08 | 0.013 |
| **MyoD** | 1.37 | 0.09 | 0.0022 |
| **CTGF** | 1.58 | 0.14 | 0.0018 |
| **RhoA** | 0.99 | 0.15 | n.s. |
| **Rock1** | 0.98 | 0.09 | n.s. |
| **Cdc42** | 0.99 | 0.09 | n.s. |
| **Rac1** | 0.99 | 0.09 | n.s. |
