# Supplementary material for: Functional regulation of YAP mechanosensitive transcriptional coactivator by Focused Low-Intensity Pulsed Ultrasound (FLIPUS) enhances proliferation of murine mesenchymal precursors
Source: PLoS One. 2018 Oct 26;13(10):e0206041. doi: 10.1371/journal.pone.0206041 (PMC6203358; doi:10.1371/journal.pone.0206041)
Supplement: S6 Table — Each stimulated value is normalized to its own unstimulated control for every time point. (DOCX) [file pone.0206041.s010.docx]

| **Time Point** | **AREG** | | | **Cyr61** | | | **CyclinD1** | | |
| --- | --- | --- | --- | --- | --- | --- | --- | --- | --- |
|  | **Mean** | **SD** | ***p*-value** | **Mean** | **SD** | ***p*-value** | **Mean** | **SD** | ***p*-value** |
| **0 h** | 0.95 | 0.06 | n.s. | 1.02 | 0.09 | n.s. | 0.99 | 0.08 | n.s. |
| **1 h** | 0.86 | 0.21 | n.s. | 1.18 | 0.06 | 0.006 | 0.97 | 0.06 | n.s. |
| **3 h** | 0.95 | 0.11 | n.s. | 0.94 | 0.05 | n.s. | 0.94 | 0.14 | n.s. |
| **5 h** | 1.20 | 0.06 | 0.0049 | 1.18 | 0.11 | 0.043 | 1.06 | 0.05 | n.s. |
| **7 h** | 1.07 | 0.05 | n.s. | 1.06 | 0.04 | 0.0019 | 1.22 | 0.07 | 0.0067 |
| **Time Point** | **ANLN** | | | **Diaph1** | | | **Diaph3** | | |
|  | **Mean** | **SD** | ***p*-value** | **Mean** | **SD** | ***p*-value** | **Mean** | **SD** | ***p*-value** |
| **0 h** | 0.93 | 0.05 | n.s. | 1.03 | 0.06 | n.s. | 0.91 | 0.05 | n.s. |
| **1 h** | 1.27 | 0.10 | 0.009 | 1.04 | 0.15 | n.s. | 1.36 | 0.02 | 9.04E-06 |
| **3 h** | 0.97 | 0.12 | n.s. | 1.05 | 0.06 | n.s. | 0.97 | 0.16 | n.s. |
| **5 h** | 1.15 | 0.02 | 0.0005 | 1.29 | 0.18 | n.s. | 1.16 | 0.02 | 8.4E-05 |
| **7 h** | 1.10 | 0.06 | 0.05 | 1.42 | 0.08 | 0.0008 | 1.05 | 0.02 | n.s. |
| **Time Point** | **CTGF** | | | **MyoD** | | | **Rock1** | | |
|  | **Mean** | **SD** | ***p*-value** | **Mean** | **SD** | ***p*-value** | **Mean** | **SD** | ***p*-value** |
| **0 h** | 1.09 | 0.27 | n.s. | 0.93 | 0.19 | n.s. | 0.96 | 0.04 | n.s. |
| **1 h** | 0.97 | 0.03 | n.s. | 1.04 | 0.29 | n.s. | 0.95 | 0.13 | n.s. |
| **3 h** | 0.97 | 0.16 | n.s. | 0.98 | 0.29 | n.s. | 1.01 | 0.13 | n.s. |
| **5 h** | 0.87 | 0.03 | 0.0024 | 0.72 | 0.14 | 0.026 | 1.12 | 0.05 | 0.013 |
| **7 h** | 1.04 | 0.09 | n.s. | 0.98 | 0.13 | n.s. | 1.09 | 0.01 | 3.0E-04 |
| **Time Point** | **CDC42** | | | **RhoA** | | | **Rac1** | | |
|  | **Mean** | **SD** | ***p-*value** | **Mean** | **SD** | ***p*-value** | **Mean** | **SD** | ***p*-value** |
| **0 h** | 0.89 | 0.10 | n.s. | 1.01 | 0.12 | n.s. | 0.99 | 0.11 | n.s. |
| **1 h** | 1.00 | 0.14 | n.s. | 0.99 | 0.12 | n.s. | 0.89 | 0.18 | n.s. |
| **3 h** | 1.04 | 0.01 | 0.0095 | 1.01 | 0.10 | n.s. | 1.08 | 0.01 | 0.0001 |
| **5 h** | 1.16 | 0.06 | 0.011 | 1.01 | 0.08 | n.s. | 1.23 | 0.07 | 0.0046 |
| **7 h** | 0.98 | 0.10 | n.s. | 1.01 | 0.04 | n.s. | 1.15 | 0.09 | 0.046 |
